# L1CAM expression as a predictor of platinum response in high‐risk endometrial carcinoma

**DOI:** 10.1002/ijc.34035

**Published:** 2022-05-10

**Authors:** Chiara Romani, Davide Capoferri, Casper Reijnen, Silvia Lonardi, Antonella Ravaggi, Martina Ratti, Mattia Bugatti, Laura Zanotti, Germana Tognon, Enrico Sartori, Franco Odicino, Stefano Calza, Johanna M. A. Pijnenborg, Eliana Bignotti

**Affiliations:** ^1^ Department of Medical and Surgical Specialties, Radiological Sciences and Public Health University of Brescia Brescia Italy; ^2^ Department of Clinical and Experimental Sciences University of Brescia Brescia Italy; ^3^ Department of Radiation Oncology Radboud University Medical Center Nijmegen The Netherlands; ^4^ Department of Pathology ASST Spedali Civili of Brescia Brescia Italy; ^5^ Division of Obstetrics and Gynecology ASST Spedali Civili di Brescia Brescia Italy; ^6^ Unit of Biostatistics and Bioinformatics, Department of Molecular and Translational Medicine University of Brescia Brescia Italy; ^7^ Department of Obstetrics and Gynaecology Radboud University Medical Center Nijmegen The Netherlands

**Keywords:** high‐risk endometrial cancer, L1CAM, platinum‐based adjuvant treatment, precision medicine

## Abstract

For high‐risk endometrial cancer (EC) patients, adjuvant chemotherapy is recommended to improve outcome. Yet, predictive biomarkers for response to platinum‐based chemotherapy (Pt‐aCT) are currently lacking. We tested expression of L1 cell‐adhesion molecule (L1CAM), a well‐recognised marker of poor prognosis in EC, in tumour samples from high‐risk EC patients, to explore its role as a predictive marker of Pt‐aCT response. L1CAM expression was determined using RT‐qPCR and immunohistochemistry in a cohort of high‐risk EC patients treated with Pt‐aCT and validated in a multicentric independent cohort. The association between L1CAM and clinicopathologic features and L1CAM additive value in predicting platinum response were determined. The effect of L1CAM gene silencing on response to carboplatin was functionally tested on primary L1CAM‐expressing cells. Increased L1CAM expression at both genetic and protein level correlated with high‐grade, non‐endometrioid histology and poor response to platinum treatment. A predictive model adding L1CAM to prognostic clinical variables significantly improved platinum response prediction (C‐index 78.1%, *P* = .012). In multivariate survival analysis, L1CAM expression was significantly associated with poor outcome (HR: 2.03, *P* = .019), potentially through an indirect effect, mediated by its influence on response to chemotherapy. In vitro, inhibition of L1CAM significantly increased cell sensitivity to carboplatin, supporting a mechanistic link between L1CAM expression and response to platinum in EC cells. In conclusion, we have demonstrated the role of L1CAM in the prediction of response to Pt‐aCT in two independent cohorts of high‐risk EC patients. L1CAM is a promising candidate biomarker to optimise decision making in high‐risk patients who are eligible for Pt‐aCT.

## Graphical abstract

What's new?

Endometrial cancer patients with high‐risk disease typically receive adjuvant platinum‐based chemotherapy. So far, no biomarkers can predict platinum chemotherapy response, one of the most important factors affecting prognosis. Here, the authors report their findings that overexpression of the transmembrane protein L1CAM predicts poor response to platinum‐based chemotherapy in high‐risk endometrial cancer patients. Inhibiting L1CAM sensitised cultured cells to carboplatin, supporting a mechanistic link between L1CAM expression and response to platinum therapy. Assessment of L1CAM could therefore improve the treatment selection process, allowing patients who are less likely to benefit from platinum to pursue alternative treatment options.

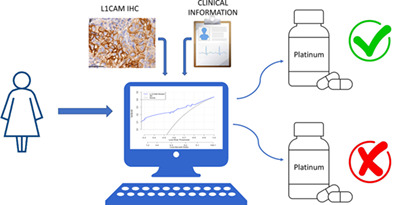

**Abbreviations**
ACME: average mediated effect
ADE: average direct effect
CSC: cancer stem cell
DSS: disease‐specific survival
EC: endometrial cancer
ENITEC: European Network for Individualised Treatment of Endometrial Cancer
FFPE: formalin‐fixed paraffin‐embedded
H&E: haematoxylin‐eosin
IDI: integrated discrimination improvement
IHC: immunohistochemistry
L1CAM: L1 cell‐adhesion molecule
LNM: lymph node metastasis
NB: net benefit
NRI: net reclassification index
OR: odds ratios
PFI: platinum‐free interval
PFS: progression‐free survival
Pt‐aCT: platinum‐based chemotherapy
Pt‐r: platinum‐resistant
Pt‐s: platinum‐sensitive
RIN: RNA integrity number
RT‐qPCR: real‐time quantitative reverse transcription PCR
STR: short tandem repeat

## 1 INTRODUCTION

Endometrial cancer (EC) is the most common malignancy of the female genital tract in developed countries, with increasing incidence due to obesity and advanced life expectancy.^1^, ^2^ Most patients are diagnosed at an early stage and have a favourable prognosis with surgery alone or in combination with adjuvant radiotherapy. However, approximately 15% to 20% of patients present with high‐risk disease characterised by: non‐endometrioid histology, high‐grade, advanced FIGO stage and extensive lymphovascular space invasion (LVSI). For those patients, adjuvant platinum‐based chemotherapy is commonly recommended and response to this systemic treatment is one of the most important factors affecting prognosis. Recently, a new classification of EC in four groups with different progression‐free survival was defined by The Cancer Genome Atlas, based on the genomic profile of tumours^3^ and subsequently translated in pragmatic assays.^4^, ^5^ While this prognostic molecular classification is becoming increasingly recognised, so far predictive biomarkers for response to adjuvant chemotherapy are currently lacking and treatment strategy remains based on evaluation of patient clinicopathological characteristics. Therefore, the identification of novel molecular predictive biomarkers remains an urgent clinical need.

The L1 neuronal cell‐adhesion molecule (L1CAM) is a transmembrane protein of the immunoglobulin family that has been extensively studied in EC and associated with lymph node metastasis (LNM), distant recurrence and poor outcome.^6^, ^7^, ^8^ However, the correlation between L1CAM expression and response to adjuvant platinum‐based chemotherapy in EC has not been investigated. Therefore, in the present study, we aimed to determine the impact of L1CAM expression on response to platinum‐based systemic treatment in two independent cohorts of high‐risk EC patients that received adjuvant platinum‐based chemotherapy. Moreover, we explored whether L1CAM could contribute to platinum response using siRNA‐mediated gene silencing in a newly established EC cell line.

## 2 MATERIALS AND METHODS

### 2.1 Patient cohorts

Our study was performed on 55 EC patients diagnosed and treated at the Division of Obstetrics and Gynaecology, ASST Spedali Civili di Brescia, Italy, between 2003 and 2018 (Italian cohort), that served as development cohort. An independent cohort of 93 EC patients was obtained from collaborating institutions within the European Network for Individualised Treatment of Endometrial Cancer (ENITEC) consortium, for external validation.^9^ Eligible patients had high‐intermediate and high‐risk ECs, defined according to the recent consensus risk grouping^10^ and underwent hysterectomy, bilateral salpingo‐oophorectomy and pelvic and/or para‐aortic lymphadenectomy, followed by platinum‐based adjuvant chemotherapy. Seventy‐two patients (48.6%) received concomitant radiotherapy. Based on the time interval between the end of platinum‐based chemotherapy and relapse (platinum‐free interval [PFI]), patients were classified into two groups: platinum‐sensitive (Pt‐s, PFI > 12 months) and platinum‐resistant (Pt‐r, PFI < 6 months). Clinical and pathological data were recorded from patients' files.

### 2.2 L1CAM and p53 immunohistochemical staining and scoring

The L1CAM immunohistochemistry (IHC) was performed on four‐micron‐thick Formalin‐Fixed Paraffin‐Embedded (FFPE) tissue sections using a 1:100 diluted monoclonal L1CAM antibody (purified anti‐CD171 [L1] antibody clone 14.10, Biolegend, San Diego, CA), according to a protocol previously described.^9^ Immunoreactivity was evaluated by two independent investigators (RC and LS), blinded for clinicopathological characteristics. L1CAM was scored according to the percentage of positive membranous staining in tumour cells (ranging from 0% to 100%) and its positive cut‐off value was set at 10%, following an established scoring system.^7^, ^9^ In case of discrepant scoring results, a consensus meeting with a third investigator (BM) was used to resolve discordancy by reviewing the slides.

For p53 IHC, a 1:100 diluted antibody clone DO‐7 (Thermo Fisher Scientific, Waltham, MA) was used, following the standard protocol applied for clinical purposes. Complete loss of expression in tumour cells (with a positive internal control in the form of positive nuclear staining of a proportion of lymphoid, endothelial, and stromal cells) and strong positive nuclear staining of >80% of tumour cells were considered as aberrant/mutation‐type (p53 abn). Normal or wild‐type pattern was characterised by heterogeneous p53 staining, commonly in the form of weak nuclear positivity in few tumour cells.

### 2.3 Tissue collection and processing

Tumour tissues were obtained from 55 patients at the time of primary surgery, snap‐frozen in liquid nitrogen within 30 minutes and stored at −80°C until processing. Tumour content was assessed by haematoxylin‐eosin (H&E) staining on frozen sections. Only samples containing at least 70% of tumour epithelial cells, as assessed by a staff pathologist, were used for further RNA extraction.

RNA was extracted using TRIZOL reagent (Life Technologies, Carlsbad, CA) and purified using RNeasy MiniElute Cleanup kit (Qiagen, Hilden, Germany) according to the manufacturer's instructions. RNA integrity was assessed with an RNA 6000 Nano LabChip kit and an Agilent 2100 Bioanalyzer (Agilent Technologies, Palo Alto, CA). RNA integrity number (RIN), generated with Agilent 2100 Expert software, was greater than 8 for all RNA samples.

### 2.4 EC cell line

Primary USC‐BS2 cell line was derived in our laboratory from a fresh clinical sample of a patient harbouring stage IV uterine serous carcinoma. Source‐patient characteristics and cell line characterisation (short tandem repeat [STR] DNA profiling, growth rate analysis and platinum sensitivity) are described in Supporting Information Materials and Methods, Tables S1 and S2 and Figures S1‐S3. Cells were maintained at 37°C in 5% CO_2_ in RPMI‐1640 medium supplemented with 10% FBS. All experiments were performed with mycoplasma‐free cells that have been authenticated using STR profiling in October 2021.

### 2.5 L1CAM gene silencing using siRNA

L1CAM Silencer Predesigned siRNA (AM16708) and Silencer Negative Control siRNA #1 (AM4611; Thermo Fisher Scientific) were employed for in vitro transient L1CAM gene knockdown. The cells were seeded onto six‐well plates and grown to 70% confluence for 48 hours before transfection with either L1CAM‐specific siRNA or negative control, using Lipofectamine2000 in Opti‐MEM medium (Thermo Fisher Scientific), according to manufacturer's instructions. After 24 hours siRNA transfection, cells were placed in fresh culture medium. For more details on gene silencing, see Supporting Information Methods and Figures S4 and S5.

### 2.6 L1CAM mRNA quantification by RT‐qPCR

cDNA was generated from total RNA extracted from both tissues and cells using the SuperScriptII Reverse Transcriptase (Invitrogen Corporation, Waltham, MA). Real‐Time Quantitative Reverse Transcription PCR (RT‐qPCR) for L1CAM and the reference gene PPIA was established in a multiplex procedure for simultaneous amplification of each template, as described by our group.^11^, ^12^ The 2^−ΔΔCt^ method was applied to calculate L1CAM relative expression. TaqMan Gene Expression Assay for L1CAM (ID: Hs01109748_m1) was obtained from Applied Biosystems as Assay‐on‐Demand product.

### 2.7 Western blot assay

Whole‐cell lysates were prepared in RIPA lysis buffer (Thermo Fisher Scientific) with protease inhibitors. Proteins were separated by SDS‐PAGE and probed with anti‐L1CAM (Clone UJ127.11, 1:2000 dilution, Sigma‐Aldrich, St. Louis, MO) and anti‐β‐tubulin (Clone B‐5‐1‐2, 1:5000 dilution, Sigma‐Aldrich) specific antibodies, followed by HRP‐labelled secondary antibody (Santa Cruz Biotech, Heidelberg, Germany). Chemiluminescent signal was acquired by iBright200 Imaging System (Thermo Fisher Scientific). Images were analysed by ImageJ‐analysis software.

### 2.8 Platinum sensitivity assay

After 24 hours siRNA transfection, USC‐BS2 cells were seeded in 96‐well plates at 5000 cells per well and allowed to attach overnight. For platinum sensitivity assay, cells were treated 48 hours post transfection in quintuplicate with seven serial dilutions of carboplatin (Sigma‐Aldrich). Drug‐free controls were included in each assay. After 72 hours, cell viability was determined by CellTiter 96 AQueous One Solution Cell Proliferation Assay (MTS; Promega Corporation, Madison, WI), according to manufacturer's instructions. Each experiment was repeated a minimum of three times. The effect of drugs on cell growth inhibition was assessed as per cent cell viability, where vehicle‐treated cells were taken as 100% viable.

### 2.9 Statistical analysis

Data were described using mean and SD for quantitative variables, counts and percentages for categorical variables.

Comparisons for L1CAM mRNA RT‐qPCR, expressed on log_2_ scale, were performed using *t*‐test, while L1CAM IHC, coded as (>10%, ≤10%), was evaluated using Chi‐square test.

The correlation between RT‐qPCR and IHC data was evaluated by Spearman rank correlation.

Prediction models for platinum resistance were fitted using logistic regression models including all relevant clinical variables (age, FIGO stage and grade) and additionally L1CAM expression, either as continuous RT‐qPCR values or IHC positivity. Results were reported as estimated odds ratios (OR) and corresponding 95% confidence intervals. Significance of L1CAM addition to the logistic model was evaluated using likelihood ratio test (Chi‐square test).

Model discrimination performance was evaluated using Harrell's concordance index (C‐index)^13^ and discrimination slope.^14^ C‐index gives the probability that a randomly selected subject who experienced an event (ie, chemoresistance) had a higher predicted risk score than a patient who was sensitive to treatment. Optimism adjusted C‐index was computed using bootstrapping (B = 200). Discrimination slope (or coefficient of discrimination) was defined as the slope of a linear regression of predicted probabilities of chemoresistence derived from the prediction model on the observed platinum resistance status.

Model calibration was reported with calibration plots showing the observed proportion of events associated to model's predicted risk.^15^

Association between L1CAM IHC positivity and prognosis, defined in terms of disease‐specific survival (DSS) and progression‐free survival (PFS), was evaluated using multivariable Cox models. DSS was defined as the time from surgery to death from disease or the last follow‐up, while PFS was computed as the interval between the time of surgery until the first clinical recurrence/progression. Kaplan‐Meier method was used to graphically show the survival curves.

To evaluate the potential mediation effect of chemoresistance on the relationship between L1CAM and prognosis, we performed a mediation analysis,^16^ using DSS as outcome and platinum resistance as a mediator.

The statistical significance of differences between scramble‐treated or siRNA‐treated cells was evaluated using paired *t* test.

Cell line data were downloaded by Cancer Cell Line Encyclopaedia (CCLE) website as RPKM values (https://data.broadinstitute.org/ccle/CCLE_RNAseq_genes_rpkm_20180929.gct.gz).

L1CAM values were extracted for selected cell lines based on their platinum susceptibility, defined according to Kharma et al.^17^ L1CAM expression was compared between resistant and sensitive cell lines using a *t*‐test on RPKM values after log2 transformation.

A decision curve analyses^18^ was performed comparing the L1CAM model with a reference “treat all” approach and a “treat none” alternative scenario.

All tests were two sided and assumed a 5% significance level. All analyses were performed using R (version 4.1.0).

## 3 RESULTS

### 3.1 L1CAM expression is related to clinicopathological factors and platinum resistance

Clinicopathologic characteristics of the 55 high‐risk EC patients included in the Italian cohort and their relationship with L1CAM mRNA expression are shown in Table 1.

**TABLE 1:** Clinicopathologic characteristics of the EC Italian study cohort (n = 55) associated with L1CAM mRNA expression and L1CAM IHC

Characteristics	Total	L1CAM mRNA logRQ	*P* *	L1CAM IHC ≤10%	L1CAM IHC >10%	*P* **
	n (%)	Mean (SD)		n (%)	n (%)	
Age						
<65	28 (50.9)	5.7 (2.9)	.489	17 (58.6)	11 (42.3)	.227
≥65	27 (49.1)	6.3 (3.5)		12 (41.4)	15 (57.7)	
Histology						
Endometrioid	31 (56.4)	4.8 (2.7)	**.001**	24 (82.8)	7 (27)	**<.001**
Non‐endometrioid	24 (43.6)	7.5 (3.2)		5 (17.2)	19 (73)	
Tumour grade						
G1‐G2	15 (27.3)	4.5 (2.8)	**.032**	13 (44.8)	2 (7.7)	**.002**
G3	40 (72.7)	6.5 (3.2)		16 (55.2)	24 (92.3)	
Myometrial invasion						
<50%	10 (18.2)	8.5 (1.9)	**.005**	2 (6.9)	8 (30.8)	**.022**
>50%	45 (81.8)	5.4 (3.2)		27 (93.1)	18 (69.2)	
FIGO stage						
I‐II	15 (27.3)	5.9 (3.1)	.896	7 (24.1)	8 (30.8)	.581
III‐IV	40 (72.7)	6.0 (3.3)		22 (75.9)	18 (69.2)	
LVSI						
Absent	5 (9)	7.9 (3.1)	.167	1 (3.4)	4 (15.4)	.124
Present	50 (91)	5.8 (3.2)		28 (96.6)	22 (84.6)	
Response to platinum treatment						
Sensitive	31 (56.4)	4.9 (2.9)	**.004**	22 (75.9)	9 (34.6)	**.002**
Resistant	24 (43.6)	7.4 (3.1)		7 (24.1)	17 (65.4)	
Recurrence						
No	22 (40.0)	5.0 (2.8)	.057	16 (55.2)	6 (23.1)	.015
Yes	33 (60.0)	6.6 (3.3)		13 (44.8)	20 (76.9)	
Death of disease						
No	27 (49.1)	5.2 (2.9)	.070	18 (62.1)	9 (34.6)	.042
Yes	28 (50.9)	6.7 (3.4)		11 (37.9)	17 (65.4)	

*Note*: L1CAM mRNA expression and IHC score are displayed. mRNA values are given as normalised relative quantification (logRQ) with SD. Significant comparisons are indicated by bold font.

^a^ *t*‐test.

^b^ Chi‐square test.

High‐grade tumours showed increased L1CAM expression (mean logRQ 6.5 vs 4.5, *P* = .032), as well as non‐endometrioid ECs (mean logRQ 7.5 vs 4.8, *P* = .001). High L1CAM expression was significantly correlated with <50% myometrial invasion (mean logRQ 8.5 vs 5.4, *P* = .005). Of note, L1CAM levels increased significantly in patients experiencing platinum resistance (mean logRQ 7.4 vs 4.9, *P* = .004), suggesting an association between this marker and response to platinum treatment. No significant difference in L1CAM mRNA expression was observed according to FIGO stage (mean logRQ 6.0 vs 5.9, *P* = .896).

**FIGURE 1:**
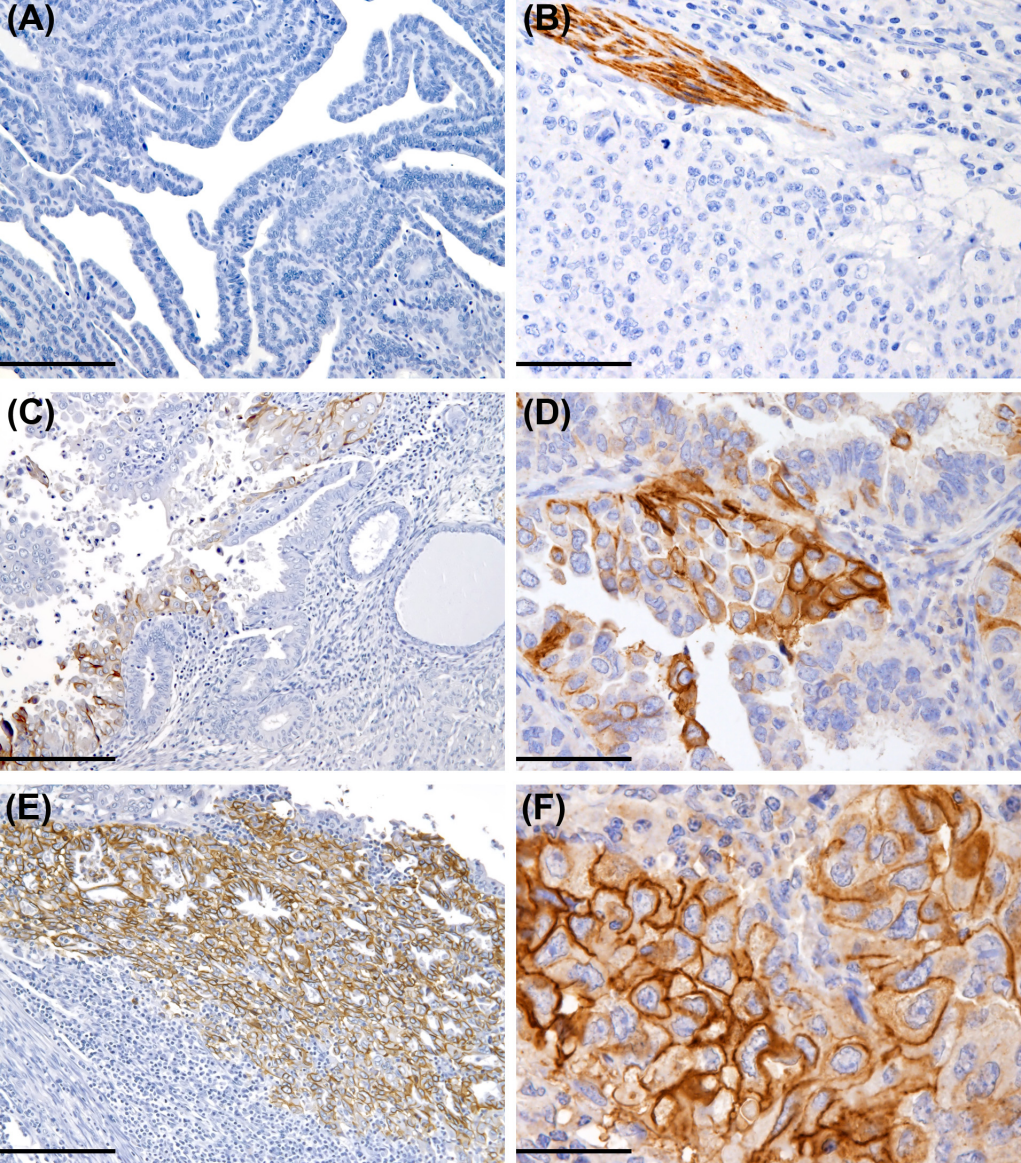
IHC staining for L1CAM in EC samples. Six representative cases with different L1CAM expressions: a serous EC (A) and an undifferentiated EC (B) negative for L1CAM, with positive staining control represented by nerves; two serous ECs (C, D) showing moderate L1CAM expression (10% of neoplastic cells); a clear cell EC (E) and a serous EC (F), showing strong membranous L1CAM staining pattern in the majority of neoplastic cells. Original magnification: ×100 (A, C, E), ×200 (B), ×400 (D, F), scale bar 200, 100 and 50 μm, respectively [Color figure can be viewed at wileyonlinelibrary.com]

Confirmation of mRNA findings at the protein level was performed by IHC staining on matched FFPE specimens, scoring L1CAM expression as reported in previous studies.^7^, ^9^ According to the established cut‐off value of >10%, 26 (47%) tumours were L1CAM positive, with 14 (54%) samples showing intense and diffuse staining in more than 50% of tumour cells (Figure 1). Significant positive association with non‐endometrioid histology, high‐grade, myometrial invasion and reduced response to platinum treatment was confirmed for L1CAM positive staining >10% (Table 1).

We found a significant association between L1CAM mRNA expression and IHC score, with Pearson's *r* = .70, indicating a high degree of concordance between the two techniques (*P* < .001).

### 3.2 L1CAM is an independent predictor for platinum response

To address the role of L1CAM in predicting platinum response, we first built a logistic model, which included the clinical variables known to be of prognostic impact in EC. Tumour grade, age and FIGO stage (hereafter “Baseline model”) showed a significant impact on predicting platinum response (Table S3), with an overall optimism adjusted C‐index of 73.5%. When adding L1CAM gene expression to the model (L1CAM model), a significant improvement in predicting platinum response was observed by means of a classical Likelihood Ratio Test (LRT), with an optimistic adjusted C‐index increasing up to 78.1% (*P* = .012).

**FIGURE 2:**
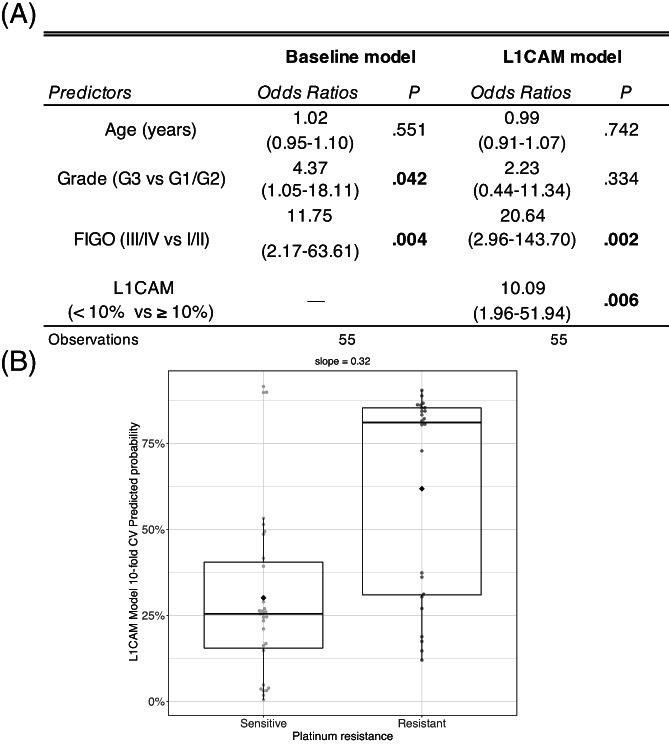
Prediction model on Italian cohort (IHC score) (A); Box plots of 10‐fold cross‐validated predicted probabilities by the BS‐L1CAM model (B). The discrimination slope (0.32) is calculated as the difference between the mean predicted probability of the truly platinum resistant vs the sensible patients (black diamonds indicate mean values)

A ROC curve for L1CAM mRNA indicated an optimal RQ threshold (computed with Youden method) value of 5.9 to discriminate patients into platinum sensitive or resistant, providing a 71.0% (95% CI: 54.8%‐87.1%) specificity and a 66.7% (95% CI: 45.8%‐83.3%) sensitivity. More than 86% of the samples above the optimal RQ threshold had an IHC score of >10% L1CAM expression. The addition of the L1CAM IHC score to the “Baseline model”, confirmed the additional predictive value for “*L1CAM model*” (LRT test *P*‐value = .002), with an optimism adjusted C‐index increasing up to 78.7% (Figure 2A). Both *Baseline* and extended with *L1CAM model* show a good calibration (correspondence between predicted probabilities and estimated from the model), with a mean absolute error of approximately 0.04 (Figure S6). Overall, these results indicate that L1CAM expression is independently associated with platinum resistance, while adjusted for all other clinical variables, and improves the performance of the prediction model. In detail, adding L1CAM to the “baseline model” yielded an increased probability of platinum resistance in 75% of true resistant patients and a decrease in 71% of true sensitive ones (net reclassification index [NRI]: 0.92, 95% CI: 0.45‐1.39, *P* < .001). Mean change of probability of platinum resistance was 7.8% higher in truly resistant (sensitivity), while was 6.1% smaller in truly sensitive (specificity), which translated in a significant Integrated Discrimination Improvement (IDI, *P* = .005).^19^ Figure 2B shows the 10‐fold cross‐validated probabilities of platinum resistance as a function of true resistance status, showing a good separation (discrimination slope = 0.32).

Moreover, we examined the performance of the model considering the cut‐off value of 50% for L1CAM positivity. The use of this higher threshold is justified by previous observations in high‐risk endometrial cancer, in which L1CAM expression >50% proved to be more appropriate in discriminating patients with a higher rate of distant metastasis.^20^

Of note, using L1CAM cut‐off >50% for discriminating platinum resistance results in an overall performance comparable to the 10% threshold in our cohort of patients (Table S4).

### 3.3 L1CAM model confirms its ability to predict platinum response in an independent EC cohort

We validated the ability of L1CAM to predict platinum response in an independent cohort of high‐risk EC patients obtained from the ENITEC consortium, matched for clinicopathologic characteristics with the Italian cohort. IHC staining was previously performed in all 93 cases using >10% as the threshold for defining L1CAM positivity.^9^ Forty nine (53%) tumours were classified as L1CAM positive, similar to the 47% positive cases in the Italian cohort and significantly associated with non‐endometrioid histology, high‐grade, advanced age and reduced response to platinum‐based chemotherapy treatment (Table 2). When the “L1CAM model” was applied to this external study cohort, a high optimism adjusted C‐index (72%, 61%‐82%) was observed. Comparison of probabilities predicted by *L1CAM model* between true sensitive and resistant patient (Figure S7) showed a good separation with a discrimination slope of 0.26. Calibration plot (Figure S8) showed a good agreement between observed and predicted proportions of event (resistant patients) with a substantial over‐estimation of risk around the 75% range.

**TABLE 2:** Clinicopathologic characteristics of high‐risk EC ENITEC validation cohort (n = 93) in relation to L1CAM positivity

Characteristics	Total	L1CAM IHC ≤10%	L1CAM IHC >10%	*P* *
	n (%)	n (%)	n (%)	
Age				
<65	44 (47.3)	28 (63.6)	16 (32.7)	**.003**
≥65	49 (52.7)	16 (36.4)	33 (67.3)	
Histology				
Endometrioid	47 (50.5)	33 (75)	14 (28.6)	**<.001**
Non‐endometrioid	46 (49.5)	11 (25)	35 (71.4)	
Tumour grade				
G1‐G2	22 (23.7)	17 (38.6)	5 (10.2)	**.001**
G3	71 (76.3)	27 (61.4)	44 (89.8)	
Myometrial invasion				
<50%	20 (21.5%)	10 (22.7)	10 (20.4)	.786
>50%	73 (78.5%)	34 (77.3)	39 (79.6)	
FIGO stage				
I‐II	18 (19.4)	11 (25)	7 (14.3)	.192
III‐IV	75 (80.6)	33 (75)	42 (85.7)	
LVSI				
Absent	50 (53.8)	24 (54.5)	26 (53.1)	.886
Present	43 (46.2)	20 (45.5)	23 (46.9)	
Missing				
Response to platinum treatment				
Sensitive	52 (55.9)	31 (70.5)	21 (42.9)	**.007**
Resistant	41 (44.1)	13 (29.5)	28 (57.1)	
Recurrence				
No	41 (44.1)	24 (54.5)	17 (34.7)	.054
Yes	52 (55.9)	20 (45.5)	32 (65.3)	
Death of disease				
No	61 (65.6)	36 (81.8)	25 (51.0)	**.002**
Yes	32 (34.4)	8 (18.2)	24 (49.0)	

Significant comparisons are indicated by bold font.

^a^ Chi‐square test.

Finally, considering both Brescia and ENITEC cohorts, p53 IHC was performed on FFPE sections of 91 EC tissues. Thirty‐eight out of 91 patients (42%) displayed a staining pattern consistent to the p53 abn molecular subtype. In this subset, we evaluated the predictive performance of a model considering L1CAM as well as FIGO stage and tumour grade. We observed 29 out of 38 (76%) L1CAM positive patients in the p53 abn subgroup, characterised by a higher risk of platinum resistance (OR = 2.78, 95% CI: 0.43‐24.4). Moreover, we found an increased accuracy (68% vs 63%) for the model including L1CAM vs a model with FIGO stage and tumour grade only, estimated via leave‐one‐out cross‐validation.

### 3.4 L1CAM affects prognosis through its effect on platinum resistance

**FIGURE 3:**
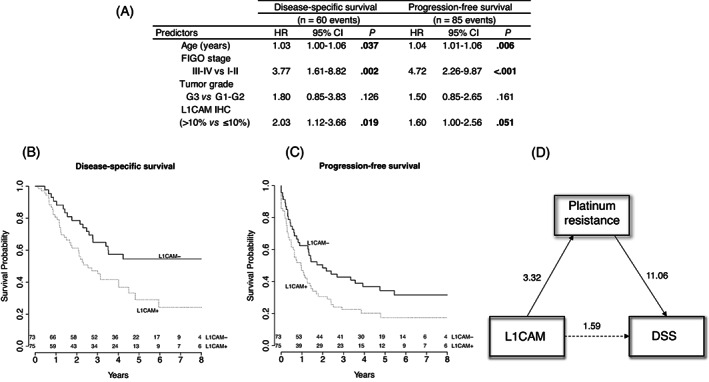
Survival analysis for all EC patients (n = 148) in the development and validation cohorts. Multivariable Cox's proportional‐hazards models (A); Kaplan‐Meier survival curves showing the L1CAM IHC effect from multivariate models adjusted to FIGO stage (III‐IV), tumour grade (G3) and age (65 years) (B, C); Mediation diagram showing the direct effect of L1CAM on DSS (HR = 1.59) and indirect with platinum resistance as mediator. Mediation analysis showed a significant average mediated effect (ACME, *P* = .018), while average direct effect (ADE) was not (*P* = .16) (D)

In multivariate survival analysis, adjusted for age, FIGO stage and tumour grade, L1CAM IHC showed a significant association with DSS (HR: 2.03, 95% CI: 1.12‐3.66, *P* = .019) and a borderline significant effect on PFS (HR: 1.60, 95% CI: 1.00‐2.56, *P* = .051); these results confirm L1CAM as an independent adverse prognostic factor (Figure 3A‐C).

To explore underlying mechanisms by which L1CAM influences survival, we estimated the direct effect of L1CAM on DSS, as well as the mediated effect by platinum resistance, through a mediation analysis. Our results suggest that the L1CAM effect on DSS is not direct, but potentially mediated by L1CAM impact on platinum resistance (Figure 3D).

### 3.5 L1CAM silencing increases platinum sensitivity in USC‐BS2 cell line

To support results obtained on clinical samples, we functionally tested the effect of L1CAM gene silencing on response to carboplatin of primary L1CAM‐expressing USC‐BS2 cells established from a platinum resistant patient.

**FIGURE 4:**
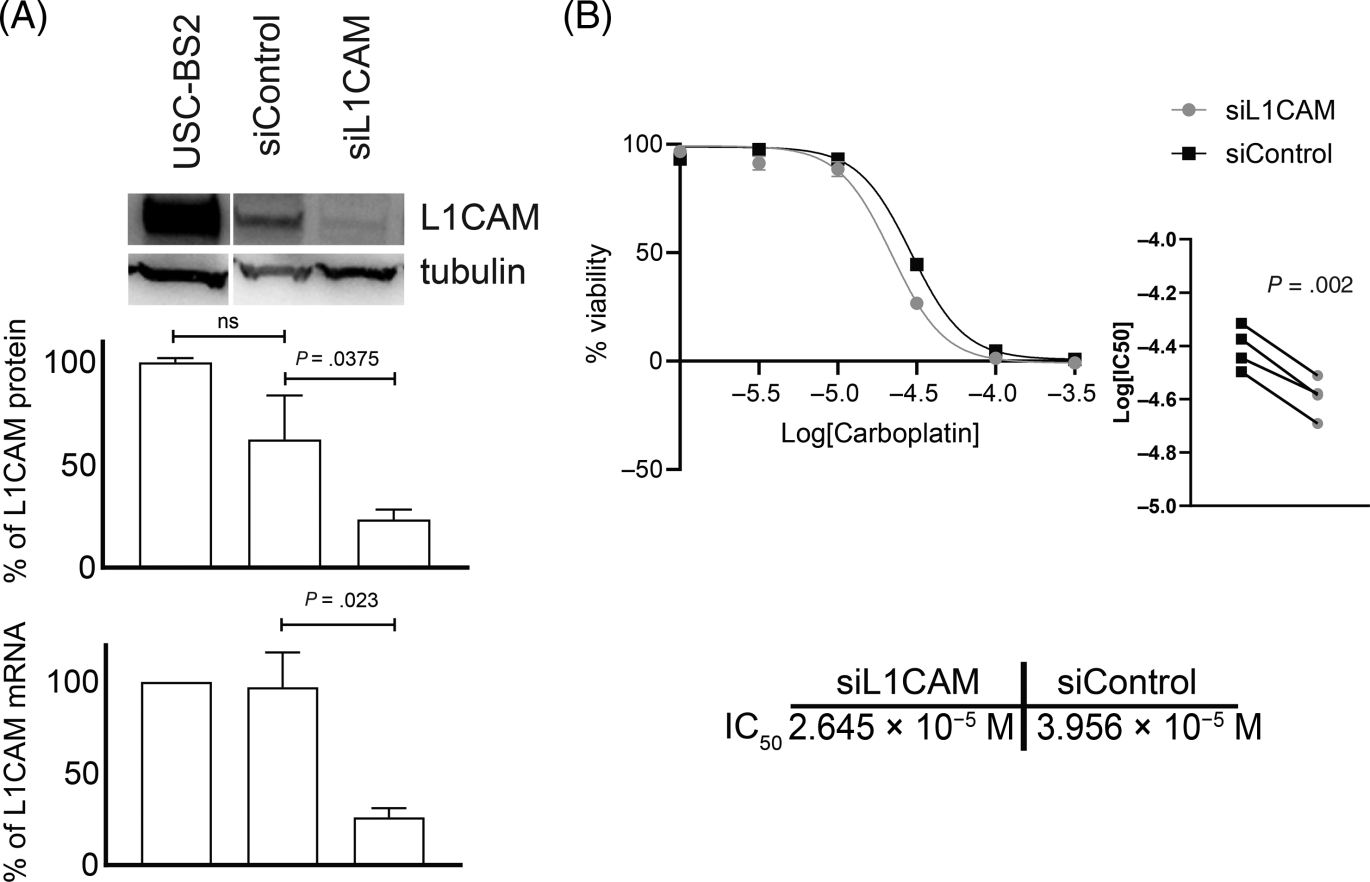
L1CAM mediates resistance to platinum in EC cells. L1CAM gene silencing by siRNA yielded a consistently significant decrease of both protein and RNA levels of 62% and 70%, respectively (A). Silenced USC‐BS2, upon 72 hours treatment with carboplatin, showed a significantly higher sensitivity (n = 4, *P* < .01) to the chemotherapeutic drug (IC50 = 26.45 μM) compared to scrambled siRNA‐treated USC‐BS2 (IC50 = 39.56 μM) (B)

As shown in Figure 4A, L1CAM mRNA levels were significantly reduced in siL1CAM USC‐BS2 compared to scrambled siRNA (siControl) cells and parental cells. Specifically, we observed a 70% mRNA decrease in siL1CAM USC‐BS2 compared to siControl cells 48 hours post transfection, which resulted in a 62% decrease of L1CAM protein. Moreover, L1CAM mRNA silencing was stable for up to 144 hours after treatment (Supporting Information Methods). On such premises, siL1CAM and siControl USC‐BS2 cells were exposed for 72 hours to increasing concentrations of carboplatin and cytotoxicity as determined by MTS assay. We observed that the concentration of drug that inhibits cell viability by 50% (IC50) was lower in siL1CAM than in siControl cells (26.45 and 39.56 μM, *P* = .0017, Figure 4B), indicating that inhibition of L1CAM significantly increase USC‐BS2 sensitivity to carboplatin compared to control. To further strengthen this result, we queried L1CAM expression across CCLE microarray gene expression data of EC cells lines and correlated it to platinum sensitivity defined according to Kharma et al.^17^ Considering three extremely sensitive cell lines (AN3CA, JHUEM2, HEC50B) and eight resistant ones (RL952, ISHIKAWA, JHUEM, HEC108, HEC265, SNGM, TEN, HEC1A), we found a significant increase of L1CAM expression (FC = 30.6, 95% CI: 2.81‐332, *P* = .01) in platinum resistant cell lines compared to sensitive ones. Taken together, these data support a potential mechanistic link between L1CAM expression and response to platinum in EC cells.

### 3.6 L1CAM evaluation has potential impact in the clinical treatment of endometrial cancer

A decision curve for the L1CAM model was built on the Italian cohort to combine “cost” and “benefit” of platinum administration, using a well‐known decision analytic metric called Net Benefit (NB).^18^ Defining the benefit of treatment as the event of interest, the risk threshold is based on the predicted probability of being sensitive: a threshold of 0.6 means a patient is judged sensitive if the predicted probability is higher than 60%. Figure S9 shows the decision curves depicting NB as a function of cost: benefit ratio for treating all scenarios (the reference one), treating none and a threshold based on the L1CAM model.

## 4 DISCUSSION

Current standard front‐line chemotherapy for high‐risk EC is represented by carboplatin/paclitaxel doublet, on whose efficacy the prognosis of patients depends. Thus, the identification of markers of response to platinum treatment remains a critical issue.

In the present study, we demonstrated that L1CAM overexpression, evaluated both at the gene and protein level, was significantly associated with platinum resistance in a cohort of high‐risk EC patients treated with adjuvant chemotherapy. This finding is in line with Asano et al^21^ who evaluated L1CAM expression in a cohort of 161 high‐risk EC patients that all received adjuvant chemotherapy, and observed an independent predictor for recurrence in L1CAM positive patients.

Besides, our results indicate that the addition of L1CAM to a chemoresistance predictive model based on clinicopathological features significantly increases the model's performance in predicting treatment response. This translates into a better classification of patients that actually benefit from adjuvant chemotherapy, and early identification of those women who might not respond to platinum‐based treatment.

In the clinical management of EC, chemotherapy is currently recommended to all high‐risk patients based on clinicopathological characteristics, with the implicit acceptance that a non trivial proportion of patients would not benefit from platinum‐based treatment. Indeed, in the Italian cohort, 24 out of 55 treated patients did not respond to platinum (43.6%) and similarly in the validation cohort (41 out of 93, 44.1%). This “treat‐all strategy” implies that there would be no advantage in avoiding a treatment that would turn out to be ineffective in a subset of patients, therefore assuming that alternative options are lacking. Nevertheless, in recent years new therapeutic strategies emerged for EC patients that could be tailored to the molecular subgroup. The relevance of L1CAM within these molecular subgroups was evaluated by Kommoss et al, who observed that 21.5% of patients were L1CAM positive across all molecular subgroups, while this proportion increased to more than 80% within the abnP53/CNH group.^22^ As platinum‐based chemotherapy is recommended in abnP53/CNH patients according to the recently updated ESGO‐ESTRO‐ESP guidelines,^10^ performing L1CAM evaluation and its association with platinum resistance might be clinically relevant. When focusing on the subset of p53 abn patients, we observed that L1CAM positivity was frequently observed (76%) which is in line with Kommoss et al^22^ and increased the risk of platinum resistance. Though limited by the relatively small number of patients, these preliminary findings on the p53 abn subgroup reveal a potential benefit of L1CAM positivity assessment in this subtype and deserves further investigation in larger cohorts where both molecular classification and outcome to platinum‐based therapy are available. The purpose of a prediction model, based on clinical parameters and molecular markers such as L1CAM, is supportive to the medical decisions process, aiming to avoid ineffective treatment in those patients who may be resistant and to opt for alternative therapies. In terms of cost: benefit ratio, this means to balance the benefit of treating the truly sensitive, while reducing the cost of useless treatment in platinum resistant patients.

Differently from Van Gool et al,^20^ in our cohort of patients the cut‐off of 10% exhibits prognostic significance, while using 50% did not show any further advantage. This explorative analysis supports the use of the well‐established threshold of 10% L1CAM expression in determining either response to platinum‐based treatment or outcome in all EC subgroups, as suggested by Van der Putten et al.^9^

Our results highlight clinical implications of L1CAM evaluation in the management of newly diagnosed EC patients that require postoperative chemotherapy. In fact, L1CAM could be incorporated in the therapeutic decision‐making process, along with the molecular markers, according to the ProMisE subgroups.^10^, ^23^ This approach will aim to direct those women who may not benefit from platinum to alternative regimens, for instance immune checkpoint inhibitors for *POLE* mutated or mismatch repair‐deficient tumours,^24^, ^25^, ^26^ and hormonal therapy or PI3K‐AKT‐mTOR inhibitors for nonspecific molecular profile (NSMP) cancers.^27^ Besides, the high frequency of L1CAM expression in high‐risk ECs indicates a potential role as a therapeutic target against which human monoclonal antibody (MAb) approaches have been explored in various types of cancer.^28^, ^29^, ^30^ Since high L1CAM expression characterizes platinum resistant disease, there is a rationale in combining L1CAM MAbs with chemotherapy because L1CAM inactivation may re‐sensitize tumour cells to standard chemotherapy. In addition, the observation that L1CAM promotes the enrichment of immunosuppressive T cells within the tumour, contributing to immune evasion and suppression,^31^, ^32^ supports the rationale of the combination between L1CAM blockade and immunotherapy as a strategy to overcome tumour immune escape. Additional studies and clinical trials are warranted to evaluate whether L1CAM blockade, in combination with chemo or immunotherapy, may have significant clinical efficacy to improve patient survival.

Our results were validated in a multicentric independent cohort of high‐risk EC patients and confirm the well‐recognised poor prognostic value of L1CAM overexpression demonstrating that the effect on survival is not direct, but mediated by L1CAM influence on response to platinum. To further endorse this observation, we performed an in vitro study which demonstrates that siRNA‐mediated L1CAM silencing significantly increases platinum sensitivity in primary EC cells. In line with our results, L1CAM expression has been reported to contribute to platinum resistance in several cancer types,^33^, ^34^, ^35^, ^36^ mainly through the modulation of intracellular signalling cascades, PI3K/Akt and MAPK pathways, affecting proliferation and apoptosis.

Recently, in colorectal carcinoma, Ganesh et al^37^ have established a link between L1CAM and cancer stemness, where L1CAM characterizes a CD133/CD44 expressing stem‐like cells population endowed with enhanced tumorigenic potential and increased chemoresistance. Moreover, L1CAM in combination with CD133 characterizes a new specific ovarian cancer stem cell (CSC) population, displaying increased radioresistance, enhanced spherogenic and clonogenic property, self‐renewal capacity and superior tumour growth in nude mice.^38^ In agreement with those observations, Chen et al^39^ has reported that L1CAM‐expressing EC cells promote epithelial‐mesenchymal transition (EMT), paclitaxel resistance and exhibit peculiar features of cancer initiating cells. Further research is needed to confirm these observations, in order to establish the rationale for assessing the impact of L1CAM‐targeted strategies on endometrial cancer CSC subpopulation. In addition, it remains unclear whether L1CAM plays a role as well in the acquired platinum resistance of initially platinum sensitive cases. We therefore strongly support future studies to evaluate L1CAM expression in tumour samples of primary and recurrent EC to further refine systemic therapy in these specific cases.

## 5 CONCLUSIONS

In our study, we demonstrate for the first time the role of L1CAM in the prediction of response to adjuvant platinum‐based chemotherapy in two independent cohorts of high‐risk EC patients. Incorporating L1CAM in the treatment decision‐process may timely identify those women who may not benefit from platinum, and require alternative treatment modalities. Moreover, L1CAM expression may be a predictive biomarker for treatment with human monoclonal antibody to resensitize tumour cells to platinum‐based chemotherapy. The L1CAM‐mediated enrichment of immunosuppressive T‐cells supports the rationale of the combination between L1CAM blockade and immunotherapy.

## AUTHOR CONTRIBUTIONS

**Eliana Bignotti, Chiara Romani, Johanna M. A. Pijnenborg:** Conceived and designed the study. **Chiara Romani, Davide Capoferri, Casper Reijnen, Silvia Lonardi, Antonella Ravaggi:** Experimental analysis. **Stefano Calza:** Statistical analysis. **Laura Zanotti, Martina Ratti, Mattia Bugatti:** Data collection. **Germana Tognon, Enrico Sartori, Antonella Ravaggi, Franco Odicino:** Analysis and interpretation of data. **Chiara Romani, Stefano Calza, Johanna M. A. Pijnenborg, Eliana Bignotti:** Writing ‐ manuscript and all authors contributed to paper discussion and reviewed the manuscript. All authors have accepted responsibility for the entire content of this manuscript and approved its submission. The work reported in the paper has been performed by the authors, unless clearly specified in the text.

## CONFLICT OF INTEREST

The authors declare no potential conflicts of interest.

## ETHICS STATEMENT

The study was performed following the Declaration of Helsinki set of principles and approved by the Research Review Board, the Ethics Committee, of the ASST‐Spedali Civili, Brescia, Italy (study reference number: NP3509). Written informed consent was obtained from all patients enrolled.

## Supporting information


**Data S1** Supporting Information

## Data Availability

The data that support the findings of our study are available from the corresponding author upon reasonable request.
