# Supplementary material for: L1CAM expression as a predictor of platinum response in high‐risk endometrial carcinoma
Source: Int J Cancer. 2022 May 10;151(4):637–48. doi: 10.1002/ijc.34035 (PMC9321598; doi:10.1002/ijc.34035)
Supplement: Supplementary file 1 — Data S1 Supporting Information [file IJC-151-637-s001.pdf]

L1CAM expression as a predictor of platinum response in high-risk endometrial carcinoma

Chiara Romani, Davide Capoferri, Casper Reijnen, Silvia Lonardi, Antonella Ravaggi, Martina Ratti, Mattia Bugatti, Laura Zanotti, Germana Tognon, Enrico Sartori, Franco Odicino, Stefano Calza, Johanna MA Pijnenborg, Eliana Bignotti

Table of contents

Supplementary Materials and Methods: Establishment and characterization of the USC-BS2 cell line; USC-BS2 cell line authentication by short tandem repeat (STR) DNA profiling; L1CAM and p53 immunohistochemical staining of USC-BS2 cell line; Growth rate analysis of the USC-BS2 cell line; Platinum sensitivity testing of the USC-BS2 cell line; L1CAM gene silencing by siRNA: evaluation of the optimal experimental setting.

Supplementary Tables: Table S1, Table S2, Table S3, Table S4.

Supplementary figures: Figure S1, Figure S2, Figure S3, Figure S4, Figure S5, Figure S6, Figure S7, Figure S8, Figure S9.

SUPPLEMENTARY MATERIALS AND METHODS

Establishment and characterization of the USC-BS2 cell line

The USC-BS2 primary culture was obtained by enzymatic-mechanical disaggregation of a fresh endometrial biopsy into single-cell suspension from a chemotherapy-naïve serous endometrial cancer patient at the time of the primary surgery [1]. Source-patient characteristics are described in Table S1. The cell lines were repetitively sub-cultured once to twice a week through 26 passage generations. The cells were maintained as a monolayer in RPMI-1640 medium supplemented with 10% FBS. Cellular growth rate was determined as described below.

USC-BS2 cell line authentication by short tandem repeat (STR) DNA profiling

USC-BS2 cell line was authenticated by short tandem repeat (STR) DNA profiling (October 2021), as described below. Genomic DNA was isolated from USC-BS2 biopsy and cells harvested at the same passage (26 th) used in *in vitro* experiments. STR profiling was performed using PowerPlex® Fusion System (Promega, Madison Corporation, WI, USA) according to the manufacturer's specifications. The amplification reaction was carried out on the thermal cycler Eppendorf™ Mastercycler™. Amplified fragments were detected by capillary electrophoresis Applied Biosystems 3130*xl* genetic analyzer. STR profiles were analyzed by GeneMapper 3.2.1 software.

A perfect concordance between STR profile of patient biopsy and the derived USC-BS2 cell line was found (Table S2). Comparison of DNA fingerprinting results in DSMZ database confirmed the unique identity of this cell line.

L1CAM and p53 immunohistochemical staining of USC-BS2 cell line

For cell block preparation, USC-BS2 cell suspensions were centrifuged for 10 minutes at 3,000 rpm. A solution of plasma (100 mL, kindly provided by Centro Trasfusionale, ASST Spedali Civili) and HemosIL RecombiPlasTin 2G (200 mL, Instrumentation Laboratory; cat. no. 0020003050; 1:2) was added to cell pellets, mixed until the formation of a clot, then placed into a bio-cassette. The specimen was fixed in 10% formalin for 1 hour followed by paraffin inclusion. The immunohistochemical stains were performed on FFPE sections from the USC-BS2 cell block following the same protocol used for L1CAM IHC in EC tissue samples, as described in the main text. P53 antibody (clone DO-7 from Agilent Technologies, dilution 1:2) was revealed using Novolink Polymer from Leica Biosystems (Wetzlar, Germany) and DAB as chromogen.

Figure S1 shows a strong positivity for both L1CAM (membranous) and p53 (nuclear).

Growth rate analysis of the USC-BS2 cell line

USC-BS2 cell line was seeded in three replicate wells per time point in 6-well plates. After 48, 72, 96, 120, 168, 192, and 216 h culture, cells were counted and assessed for viability via trypan blue exclusion (Fig. S2).

Platinum sensitivity testing of the USC-BS2 cell line

For experiments in microscale, cells were seeded in 96-well plates at appropriate density in 200 µL medium and grown for 48 hours before treating in quintuplicate with seven serial dilutions (from 300 µM to 0.3 µM, according to [2]) of carboplatin (Sigma-Aldrich, St. Louis, MO, USA). After 72 h, cell viability was determined by CellTiter 96® AQueous One Solution Cell Proliferation Assay (MTS) (Promega Corporation), according to manufacturer's instructions. The experiment was repeated three times. The effect of drugs on cell growth inhibition was assessed as percent cell viability, where vehicle-treated cells were taken as 100% viable. As shown in Fig. S3, we determined a fitting treatment curve of 3-300 µM, and we observed that the concentration of drugs that inhibited cell viability by 50% (IC50) was 59.93 µM.

L1CAM gene silencing by siRNA: evaluation of the optimal experimental setting

L1CAM Silencer Pre-designed siRNA (AM16708) and Silencer® Negative Control siRNA #1 (AM4611) (ThermoFisher Scientific, Waltham, MA, USA) were employed for in vitro transient L1CAM gene knockdown. The cells were seeded onto 6-well plates and grown to 70% confluency for 48 h before transfection with increasing concentrations (10, 30, 60, 90 pM) of either L1CAM specific siRNA or negative control, using Lipofectamine2000 in Opti-MEM medium (ThermoFisher Scientific), according to our previous work [2]. After 24 h siRNA

transfection, cells were placed in fresh culture medium and after a further 24 h, gene silencing was assessed by RT-qPCR.

A 70% reduction in L1CAM mRNA was achieved at 30 pM and 60 pM siRNA (Fig. S4), and we decided to use the lowest concentration (30 pM) for all subsequent carboplatin sensitivity assay.

SUPPLEMENTARY TABLES

Table S1 Clinical features of the patient and characteristics of the tumor used to derive USC-BS2 cell line

**Clinical Parameter**	
Age at diagnosis (y)	70
Tumor type	adenocarcinoma
Histopathology sub-type	serous
Tumor grade	G3
Disease stage	IV
Myometrial invasion	>50%
Lymphovascular invasion	yes
Proliferation index (Ki-67)	80%
p53 positivity	100%
Estrogen and progesterone receptors	minimal and focal positivity
Progression	yes
Death	yes
Cause of death	progression
Overall survival (months)	21
First-line treatment	Surgery, Cisplatin-epirubicin-paclitaxel
Platinum Free Interval (months)	0
Previous personal history of cancer	no
Year of sampling	2012
Chemotherapy naïve at sample collection	yes

Table S2 STR profiles of USC-BS2 biopsy and the derived cell line**.**

Markers	USC-BS2 cell line	USC-BS2 biopsy
**AMEL**	X	X
**D3S1358**	14	14
**D1S1656**	15.3 (16)	15.3 (16)
**D2S441**	11	11
**D10S1248**	(13) (14) 15	13 14 15
**D13S317**	11 13	11 13
**Penta E**	12	12
**D16S539**	11	11
**D18S51**	15	15 (19)
**D2S1338**	19 20	19 20
**CSF1PO**	10 12	10 12
**Penta D**	10 12	10 12
**TH01**	7	7
**vWA**	(14) 16	14 16
**D21S11**	31.2 33.2	31.2 33.2
**D7S820**	9 12	9 12
**D5S818**	11 13	11 13
**TPOX**	8 11	8 (11)
**DYS391**	-	-
**D8S1179**	11 13	11 13
**D12S391**	(22) 25	(22) 25
**D19S433**	13	13
**FGA**	20 25	20 25
**D22S1045**	16	16

( ): in round brackets, weak peaks or particularly intense stutters

Table S3. Prediction model of platinum resistance on Italian cohort (gene expression)

Predictors	Baseline Model			L1CAM Model		
	**OR**	**95% CI**	**p-value**	**OR**	**95% CI**	**p-value**
Age (years)	1.02	0.95-1.10	0.551	1.00	0.92-1.08	0.943
Grade (G3 vs G1/G2)	4.37	1.05-18.1	**0.042**	3.11	0.67-14.5	0.149
FIGO stage (III/IV vs I/II)	11.75	2.17-63.6	**0.004**	14.05	2.28-86.5	**0.004**
L1CAM RQ				1.32	1.05-1.65	**0.018**
Observation number	55			55		

OR = odds ratio. Significant p-values are in bold.

Table S4. Prediction model on Italian cohort using L1CAM IHC cut-off > 50%.

Predictors	Model		
	**OR**	**95% CI**	**p-value**
Age (years)	1.00	0.92-1.08	0.987
Grade (G3 vs G1/G2)	3.40	0.77-15.1	0.107
FIGO stage (III/IV vs I/II)	11.75	2.17-63.6	**0.004**
L1CAM (>50% vs <50%)	6.29	1.07-36.8	**0.041**
Observation number	55		

OR= odds ratio. Significant p-values are in bold.

SUPPLEMENTARY FIGURES

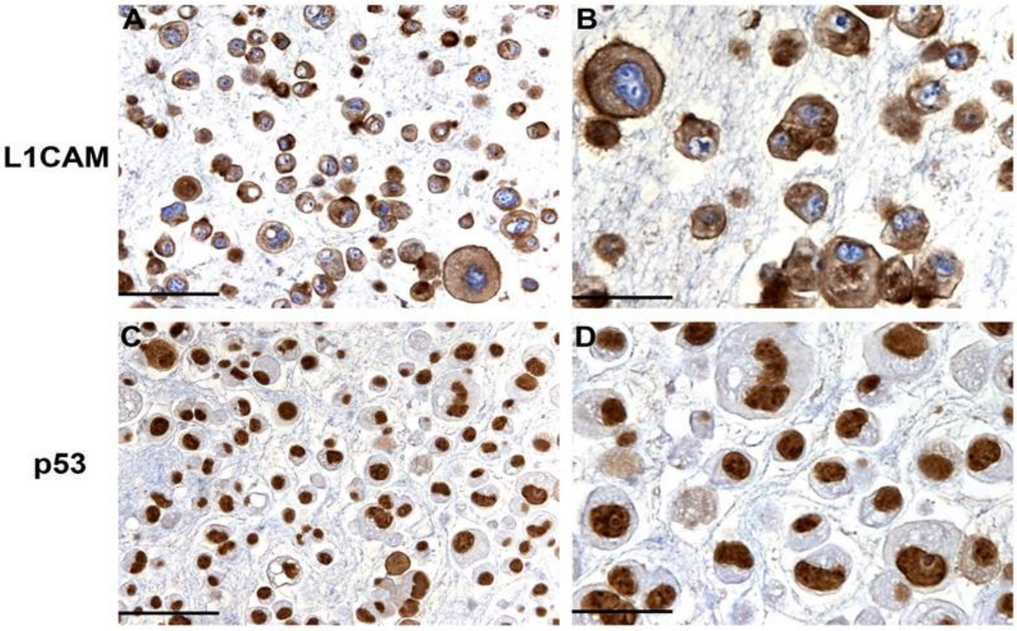

Figure S1 USC-BS2 cell line stained for L1CAM (A, B) and p53 (C, D). Original magnification of 200x (A, C) and 400x (B, D), scale bar 100 μm and 50 μm respectively.

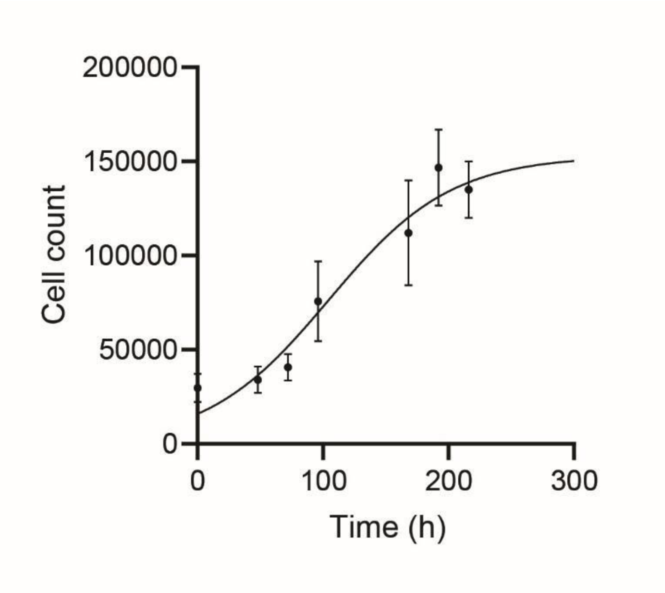

Figure S2 Proliferation curve of USC-BS2 cell line. Using a logistic growth model, we calculated a lag time of 48 hours between cell seeding and exponential growth.

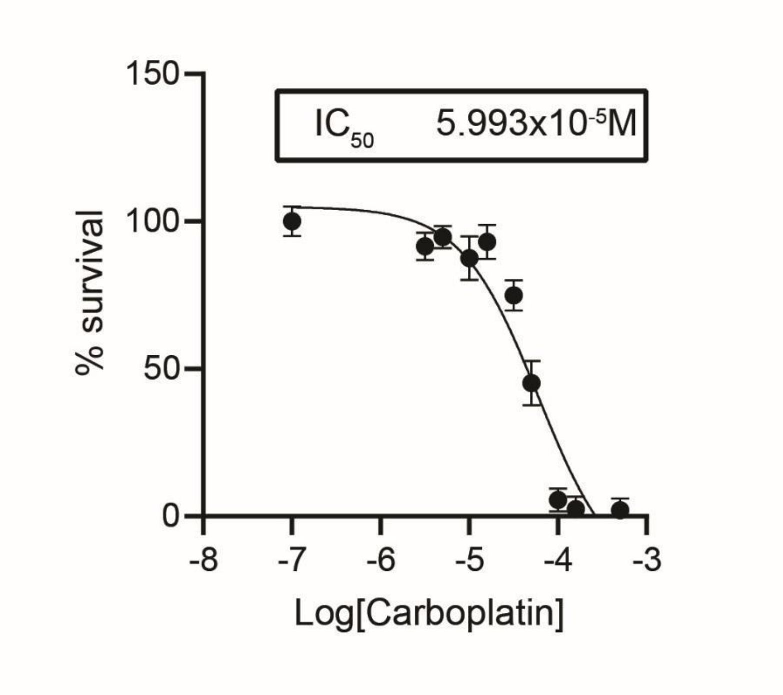

Figure S3 Dose-response curves for USC-BS2 cell line indicate the percentage of cell viability compared to untreated control and are represented as mean ± SD of three independent experiments.

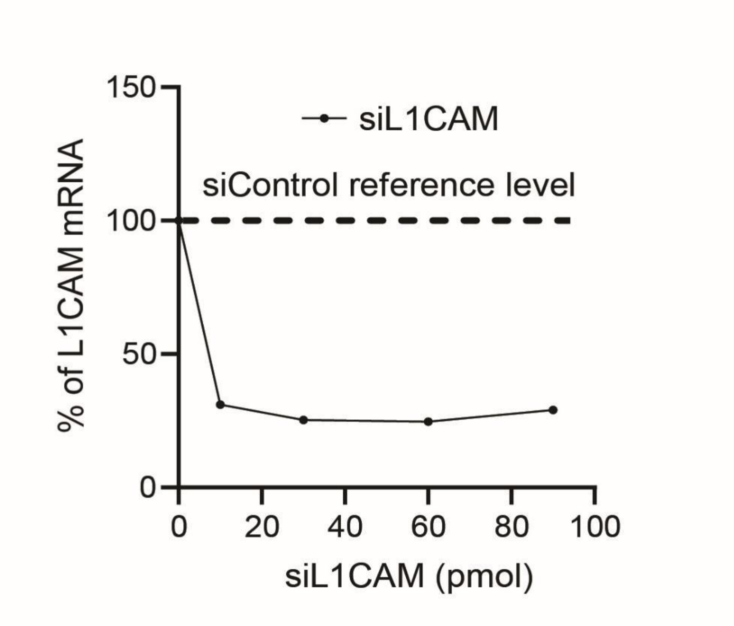

Figure S4 Dose-response curve of L1-CAM gene knockdown after L1CAM Silencer siRNA (siL1CAM) transfection compared to Silencer Negative Control siRNA (siControl).

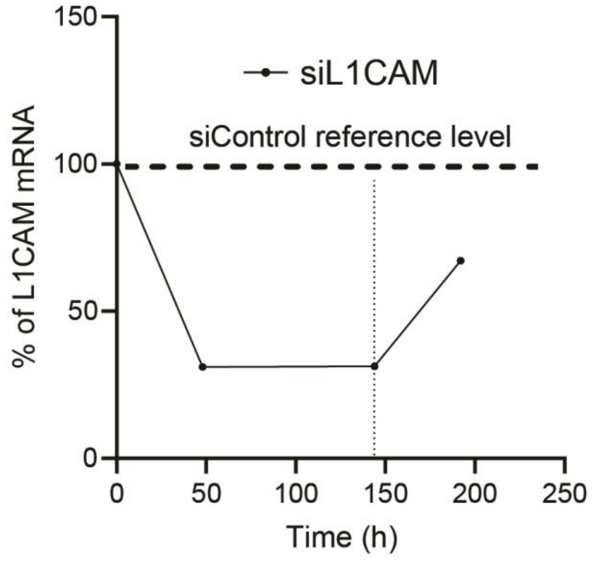

Figure S5 Stability curve of L1CAM silencing after transfection with 30 pM siRNAs.

The duration of L1CAM transient silencing was assessed on cells harvested 48, 144, and 192 hours after transfection with 30 pM siRNAs. From 48 to 144 hours, the reduction in L1-CAM mRNA was stable at 70-75%.

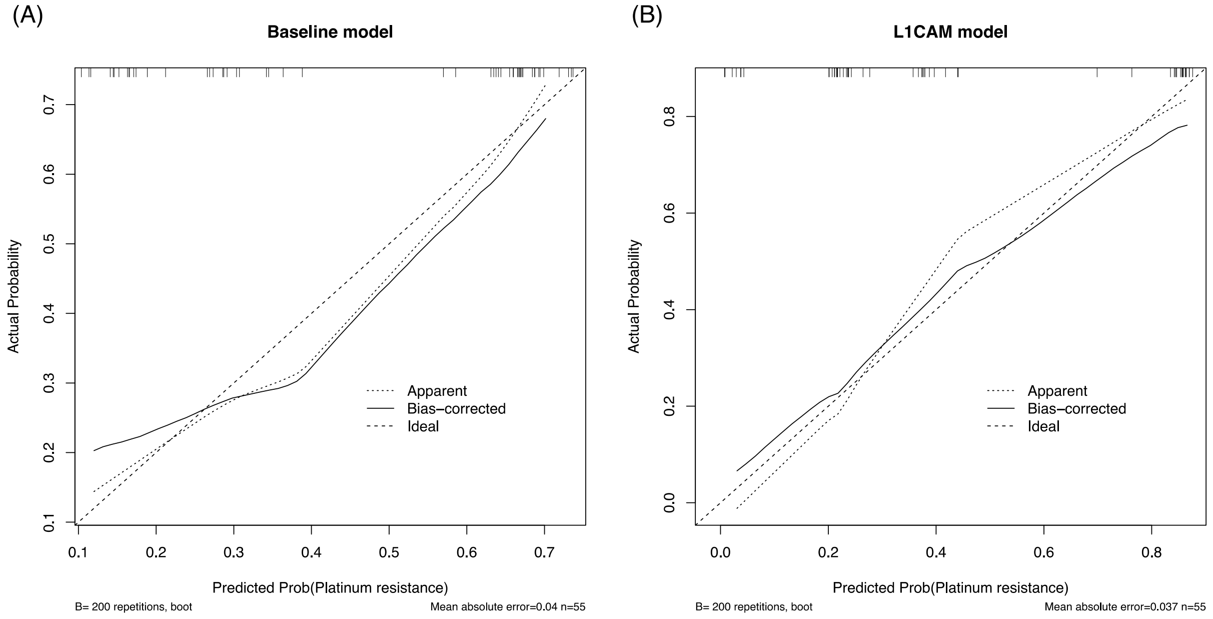

Figure S6. Calibration Plot. Calibration curve for predicted probabilities using the Baseline (A) and L1CAM (B) models in the Italian cohort. Dashed line represents the ideal relationship between predicted and observed probabilities. Solid black line represents bias corrected, while dotted line represents apparent calibration

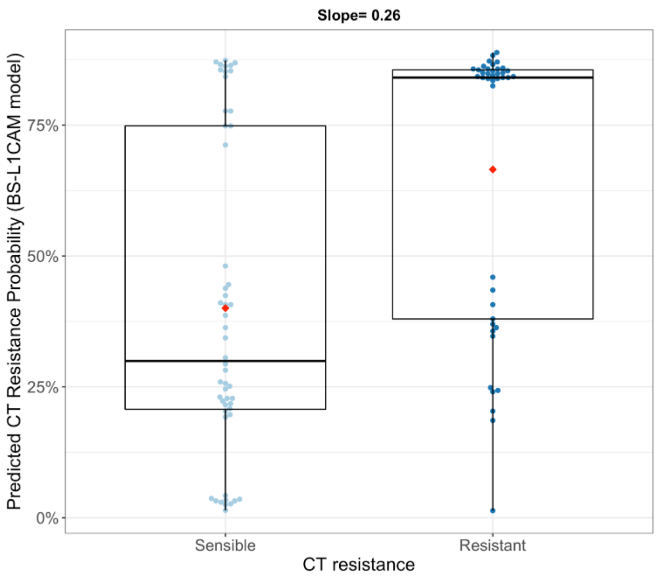

Figure S7. Box plots of predicted probabilities of platinum chemoresistance in using the L1CAM model. The discrimination slope (0.26) is calculated as the difference between the mean predicted probability of the truly platinum resistant versus the sensible patients (red diamonds indicate means).

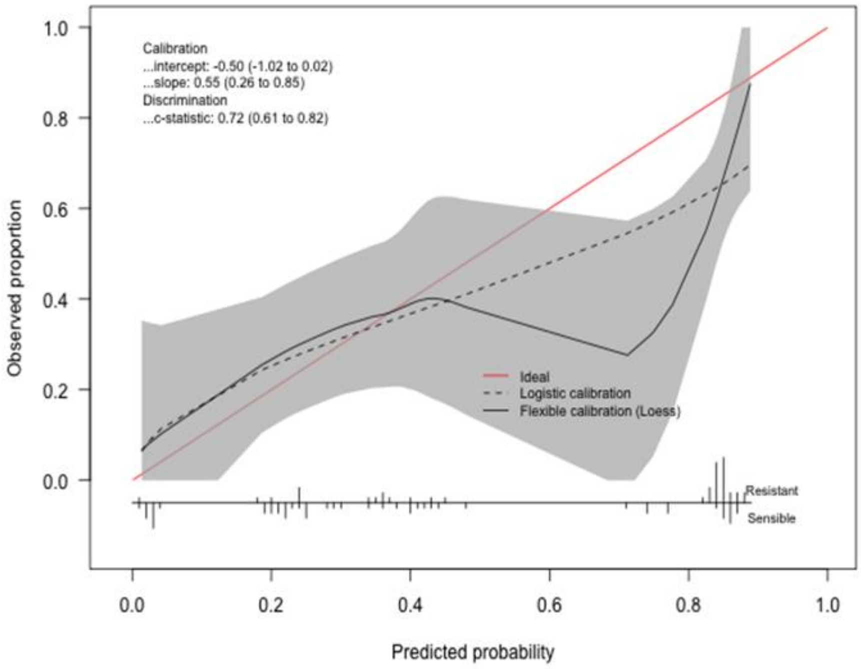

Figure S8. Calibration Plot. Calibration curve for predicted probabilities using the L1CAM model in the ENITEC cohort.

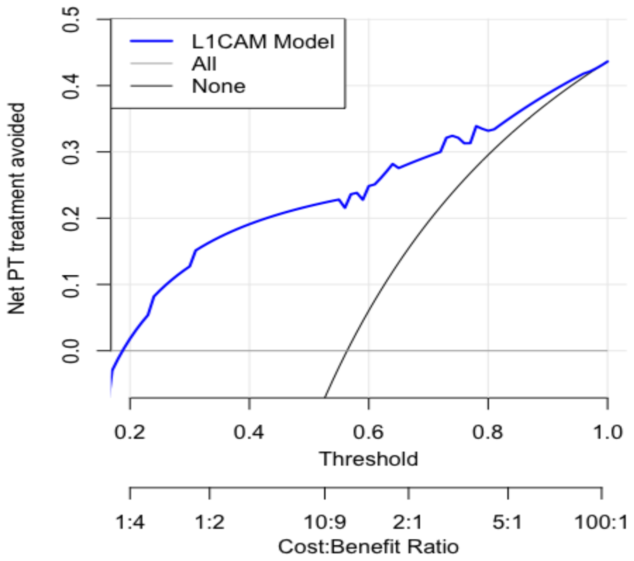

Figure S9. Decision curve for the L1CAM model fitted on the Italian cohort. Assuming a cost:benefit ratio of 2:3, which corresponds to a risk threshold of 0.4, the NB for the choice based on the L1CAM model would be 19.1%, that is approximately 19% more resistant patients would be addressed to alternative treatment schemes.

SUPPLEMENTARY REFERENCES

[1] AD Santin, F Zhan, S Cane', S Bellone, M Palmieri, M Thomas et al. Gene expression fingerprint of uterine serous papillary carcinoma: identification of novel molecular markers for uterine serous cancer diagnosis and therapy. Br J Cancer. 2005 Apr 25; 92(8): 1561–1573.

[2] RA Tassi, P Todeschini, ER Siegel, S Calza, P Cappella, L Ardighieri et al. FOXM1 expression is significantly associated with chemotherapy resistance and adverse prognosis in non-serous epithelial ovarian cancer patients. J Exp Clin Cancer Res. 2017; 36: 63.
